# Calcipotriol and iBRD9 reduce obesity in Nur77 knockout mice by regulating the gut microbiota, improving intestinal mucosal barrier function

**DOI:** 10.1038/s41366-020-0564-0

**Published:** 2020-03-17

**Authors:** Qingqing Lv, Aolin Yang, Wanying Shi, Feng Chen, Yixuan Liu, Ying Liu, Difei Wang

**Affiliations:** 1 Nutrition Department, The First Hospital of China Medical University, Shenyang, Liaoning China; 2 Department of Geriatric Endocrinology, The First Hospital of China Medical University, Shenyang, Liaoning China; 3 Department of Biochemistry and Molecular Biology, China Medical University, Shenyang, Liaoning China

**Keywords:** Diagnostics, Obesity

## Abstract

**Objective:**

The orphan nuclear receptor Nur77 is an important factor regulating metabolism. Nur77 knockout mice become obese with age, but the cause of obesity in these mice has not been fully ascertained. We attempted to explain the cause of obesity in Nur77 knockout mice from the perspective of the gut microbiota and to investigate the inhibitory effect of calcipotriol combined with BRD9 inhibitor (iBRD9) on obesity.

**Methods:**

Eight-week-old wild-type mice and Nur77 knockout C57BL/6J mice were treated with calcipotriol combined with iBRD9 for 12 weeks. Mouse feces were collected and the gut microbiota was assessed by analyzing 16S rRNA gene sequences. The bacterial abundance difference was analyzed, and the intestinal mucosal tight junction protein, antimicrobial peptide, and inflammatory cytokine mRNA levels of the colon and serum LPS and inflammatory cytokine levels were measured.

**Results:**

Calcipotriol combined with iBRD9 treatment reduced the body weight and body fat percentage in Nur77 knockout mice. In the gut microbiota of Nur77 knockout mice, the relative abundances of *Lachnospiraceae* and *Prevotellaceae* decreased, and *Rikenellaceae* increased; while *Rikenellaceae* decreased after treatment (*p* < 0.05). Correspondingly, the mRNA levels of intestinal mucosal tight junction proteins (occludin (Ocln), claudin3 (Cldn3)) in the colons of Nur77 knockout mice were significantly decreased, and they increased significantly after treatment (*p* < 0.001). The mRNA levels of inflammatory cytokines (tumor necrosis factor-α (TNF-α), interleukin-6 (IL-6), and interleukin-1β (IL-1β)) were significantly increased in Nur77 knockout mice, and TNF-α and IL-6 levels were significantly decreased after treatment (*p* < 0.05, <0.01, or <0.001). The levels of serum LPS, TNF-α, and IL-1β in Nur77 knockout mice were significantly increased (*p* < 0.05). Serum LPS, TNF-α, and IL-6 levels were significantly decreased after treatment (*p* < 0.05 or <0.01).

**Conclusions:**

Calcipotriol combined with iBRD9 can regulate the gut microbiota, improve intestinal mucosal barrier function, reduce LPS absorption into the blood, and alleviate obesity in Nur77 knockout mice.

## Introduction

The immune response is essential to protect the body from physical, chemical, and biological damage. However, persistent low-grade inflammatory conditions can cause damage to the body tissues and is the cause of metabolic diseases such as obesity, diabetes, and other chronic noncommunicable diseases [1]. Nuclear receptor subfamily 4, group A (NR4A) is an important regulator of the inflammatory response, which can directly promote the expression of FoxP3 transcription factor and is related to the production, differentiation, and maintenance of regulatory T (Treg) cells [2], and T-cell-specific deletion of all NR4A family members causes significant multiorgan inflammation [3, 4]. NR4A1 (also called Nur77) has no effect on FoxP3 transcription, and Nur77 knockout mice produce Treg cells and cannot develop autoimmune diseases [5, 6]. Nur77 knockout mice become obese with age and have low-grade inflammation [7, 8]. However, the cause of the systemic low-grade inflammation and obesity has not been fully explored.

The influence of the gut microbiota on energy metabolism has attracted considerable attention [9]; it can regulate metabolic homeostasis, and gut microbiota dysbiosis has been proven to be associated with obesity [10, 11]. Studies have shown that there is a significant difference in the composition of the gut microbiota between hereditary obese (ob/ob) mice and wild-type mice [12], and additional studies have found that the composition of the gut microbiota is affected by genotype [13–15]. Therefore, we suspect that Nur77 deficiency may cause changes in the gut microbiota and attempted to elucidate the cause of obesity in Nur77 knockout mice from the perspective of the gut microbiota.

Cross-sectional studies show that vitamin D deficiency is positively associated with obesity [16], but vitamin D supplementation does not effectively reduce obesity [17–19]. Mechanistic research shows that calcitriol increased lipolysis and energy consumption and reduced lipid content in adipocytes in vivo in part through regulation of β-oxidation and UCP expression regulated by vitamin D receptor (VDR) [20–22]. In addition, interest in the anti-inflammatory potential of vitamin D continues to grow. An interesting study showed that VDR shuttles between BAF and PBAF complexes in a ligand-dependent manner, and iBRD9 cooperates with VDR ligand to favor PBAF complex binding, which enhances chromatin accessibility at consensus VDR binding elements to modulate the expression of key inflammatory response genes [23]. The anti-inflammatory effect of vitamin D and iBRD9 combined is worthy of further research.

Therefore, we intended to use the VDR ligand calcipotriol and iBRD9 to intervene in Nur77 knockout mice to explore the effects of vitamin D on obesity in Nur77 knockout mice and investigated changes in the gut microbiota.

## Materials and methods

### Animals and treatments

Nur77 knockout mice with C57BL/6J as the background were produced in the Jackson Laboratory (Bar Harbor, ME). Fourteen male wild-type (C57BL/6J) mice that were 3 weeks old were purchased from Changsheng Biotechnology Co., Ltd. (Liaoning, China), housed in transparent plastic feeding cages (seven mice per feeding cage), kept in a controlled environment (12-h light-dark cycle, 22 ± 1 °C), and provided with unlimited food (GB 14924.3-2010 feed formula) and deionized water. All experimental protocols were approved by the Animal Care and Use Committee of China Medical University.

Seven-week-old wild-type C57BL/6J and Nur77 knockout male mice were randomly divided into two groups of seven mice each. The mice were adapted at 8 weeks of age. One group of the wild-type mice was separately injected intraperitoneally with the vehicle (30% hydroxypropyl-β-cyclodextrin), and the other group was injected with calcipotriol combined with iBRD9. The Nur77 knockout mice were treated in the same way. The four groups were the wild-type mouse vehicle control group (WT-V), wild-type mouse treatment (calcipotriol combined with iBRD9) group (WT-T), Nur77 knockout mouse vehicle control group (KO-V), and Nur77 knockout mouse treatment group (KO-T). Calcipotriol combined with iBRD9 was administered three times a week for a total of 12 weeks. The doses were [23] calcipotriol at 60 µg/kg, and iBRD9 at 10 mg/kg.

Fresh feces were collected before the mice were sacrificed, frozen in liquid nitrogen, stored in a freezer at −80 °C, and taken out before sequencing. After the mice were euthanized, blood and colon tissues were obtained. The blood was centrifuged at 3000 × *g* for 25 min at 4 °C, and the supernatant serum was collected and stored in a −80 °C freezer for use.

### Body weight and fat mass measurement

Body weights were monitored weekly. Fat mass and lean mass were determined in the last week using a Bruker Minispec LF50.

### Biochemical analyses

Mouse serum triacylglycerol (TG) and total cholesterol (TC) were measured by an enzyme colorimetric assay using a commercial assay kit (Jiancheng Bioengineering Research Institute Co., Ltd, Nanjing, China). Mouse serum leptin (LEP), TNF-α, IL-6, and IL-1β concentrations were determined using an enzyme-linked immunosorbent assay kit (Xinfan Technology Co., Ltd, Shanghai, China). A Pierce^TM^ Color Rendering Endotoxin Quantitation Kit (88282, Thermo) was used to detect lipopolysaccharide (LPS) levels in serum by using the limulus amebocyte lysate assay. A blood glucose meter (NC, Roche, Germany) was used to measure tail vein blood glucose after fasting overnight. Serum calcium (Ca) and phosphorus (P) were detected using the methyl thymol blue method and the phosphomolybdic acid method, respectively (Xinfan Technology Co., Ltd, Shanghai, China).

### Fecal 16S rRNA analysis

Total genome DNA from samples was extracted using the CTAB/SDS method. DNA concentration and purity were monitored on 1% agarose gels. According to the concentration, DNA was diluted to 1 ng/µL using sterile water. The extracted DNA from each sample was used as template to amplify the V3 + V4 region of 16S rRNA genes of distinct regions (16S V3 + V4) with specific primers (341F: 5′-CCTAYGGGRBGCASCAG-3′, 806R: 5′-GGACTACNNGGGTATCTAAT-3′). All PCR reactions were carried out in 30 µL reactions with 15 µL of Phusion^®^ High-Fidelity PCR Master Mix (New England Biolabs). PCR products were mixed with the same volume of 1× loading buffer (containing SYBR green) and detected with electrophoresis on a 2% agarose gel. PCR products were mixed in equidensity ratios. Then, the mixture of PCR products was purified with a GeneJET^TM^ Gel Extraction Kit (Thermo Scientific). Sequencing libraries were generated using Ion Plus Fragment Library Kit 48 rxns (Thermo Scientific) following the manufacturer’s recommendations. The library quality was assessed on the Qubit@ 2.0 Fluorometer (Thermo Scientific). Finally, the library was sequenced on an Ion S5^TM^ XL platform and 600 bp single-end reads were generated.

### Quantification of genes expression in colon tissue

Total RNA was isolated from ~40 mg of colon tissue using TRIzol reagent according to the manufacturer’s instructions (Life Technologies, CA) and quantified by a Nano Photometer-N50 (Implen, Germany). cDNA was synthesized from 500 ng of total RNA using an iScript cDNA Synthesis Kit (BIO-RAD, USA). Real-time quantitative PCR (qPCR) was performed using a Power Up SYBR Green master mix (Applied Biosystems, USA) and an LC480 II (Roche) qPCR instrument. The qPCR results were calculated using the 2^−ΔΔCt^ method. Primer sequences are shown in Supplementary Table 1.

### Statistical analysis

Experimenters were blind to the groups during data analysis. No animals were excluded from the analyses. Statistical analysis was performed using SPSS v17.0 (Chicago, IL, USA), and *p* < 0.05 was considered significant in all cases. Data are expressed as the mean ± standard error of the mean (mean ± SEM). For comparisons among multiple groups, one-way analysis of variance (ANOVA) and the Bonferroni post hoc test was used to compare the two groups. For 16S rRNA sequencing data, statistical tests were performed in the R programming environment [24].

## Results

### Nur77 knockout mice gain weight, and weight loss occurs after treatment with calcipotriol combined with iBRD9

**Fig. 1:**
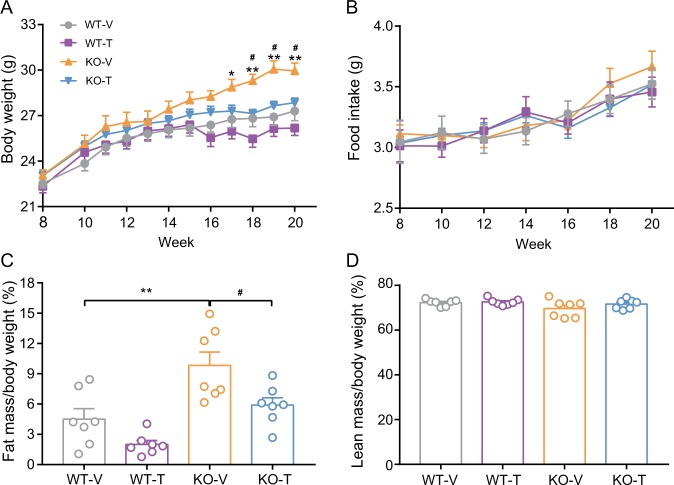
The effects of calcipotriol and iBRD9 on body weight, food intake, and body composition of Nur77 knockout mice. **a** Weight gain curves; **b** food intake curves; **c** the percentage of body fat mass; **d** the percentage of body lean mass (*n* = 7/group). Data are expressed as the mean ± SEM. *p < 0.05; **p < 0.01 versus WT-V; ^#^*p* < 0.05 versus KO-V.

After 17 weeks of age, Nur77 knockout mice showed a significant increase in body weight, which decreased significantly after treatment to a level that was close to the weight of the wild-type mice (*p* < 0.05) (Fig. 1a). However, there was no significant difference in mouse food intake (Fig. 1b). The difference in body weight was reflected mainly in body fat, as shown in Fig. 1c; Nur77 knockout mice showed a significant increase in body fat percentage that significantly decreased after treatment (*p* < 0.05 or <0.01), and there was no significant difference in the lean mass ratio in mice (Fig. 1d).

### Serum biochemical parameters of calcipotriol and iBRD9-treated Nur77 knockout mice

The LEP level was significantly increased in Nur77 knockout mice and was significantly reduced after treatment (*p* < 0.05 or <0.01). In addition, there was no significant difference in fasting blood glucose (FBG), TG, TC, Ca, and P (Supplementary Table 2).

### The diversity of gut microbiota in calcipotriol and iBRD9-treated Nur77 knockout mice

**Fig. 2:**
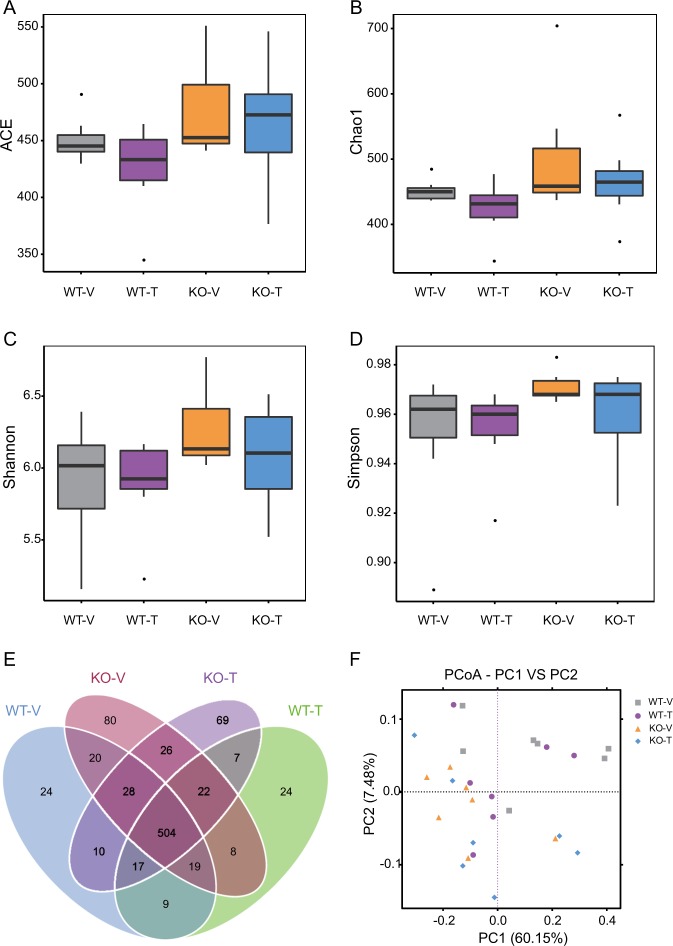
Calcipotriol and iBRD9 treatment effect on the gut microbiota structure in Nur77 knockout mice. Alpha diversity analysis: the ACE estimator (**a**), Chao1 estimator (**b**), Shannon index (**c**), and Simpson index (**d**) were used for evaluation. The results are the means ± SEM (*n* = 7). Data were analyzed by 1-factor ANOVA, followed by the Tukey–Kramer multiple comparison test. Venn diagram (**e**) showing the unique and shared OTUs in the gut microbiota between groups. Plots were generated using a weighted UniFrac distance-based PCoA (**f**).

Community richness was determined using the ACE estimator and the Chao1 estimator (Fig. 2a, b), and community diversity was estimated using the Shannon index and the Simpson index (Fig. 2c, d). Community richness and diversity were not significantly different between groups according to the Wilcoxon rank sum test.

For 99.88% of the operational taxonomic units (OTUs), based on the common and unique OTUs between the four groups, a Wayne diagram is shown in Fig. 2e. The microbial community composition of different samples was compared by principal coordinate analysis (PCoA) (Fig. 2f). A significant difference was found between KO-V and WT-V, and there was a significant difference between KO-T and KO-V(*p* < 0.05 or <0.01).

### LEfSe analysis in calcipotriol and iBRD9-treated Nur77 knockout mice

**Fig. 3:**
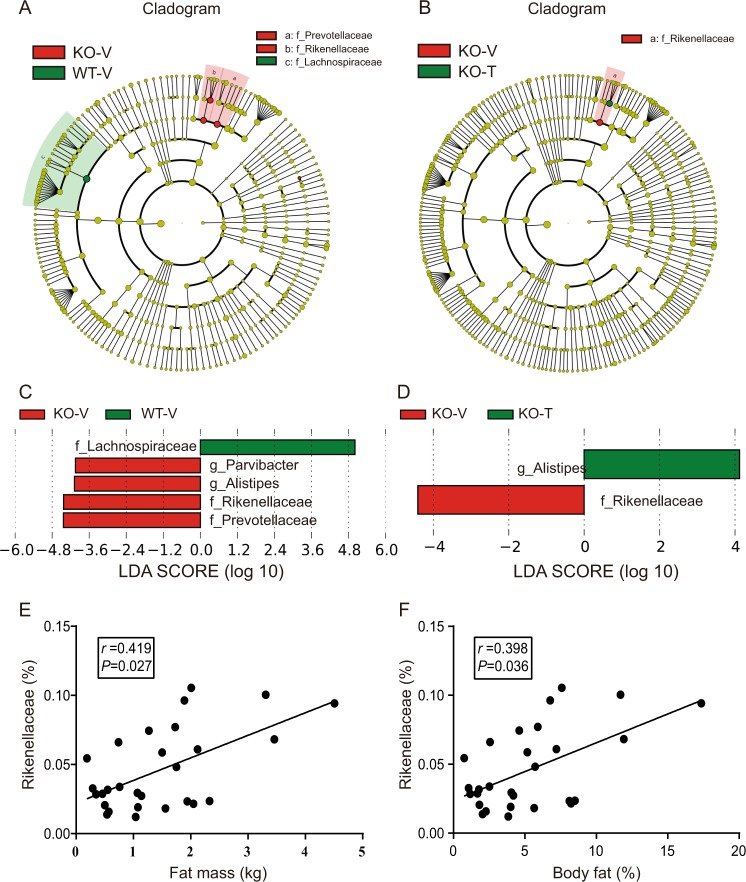
Specific biomarkers of calcipotriol and iBRD9-treated Nur77 knockout mice. **a**, **b** Cladogram representation of the differentially abundant families and genera. The root of the cladogram denotes the domain bacteria. The taxonomic levels of the phylum and class are labeled, while family and genus are abbreviated, with the colors indicating the greatest abundance. The size of each node represents their relative abundance. **c**, **d** LEfSe analysis shows differentially abundant genera as biomarkers determined using the Kruskal–Wallis test (*p* < 0.05) with an LDA score > 4. Correlation between the relative abundance of *Rikenellaceae* and biological parameters after calcipotriol and iBRD9 treatment. **e** Fat mass (kg) and **f** body fat (%) exhibited significant correlations with levels of *Rikenellaceae* in the fecal microbiota. *p* < 0.05 based on Spearman rank correlation analysis, *n* = 28.

Linear discriminant analysis (LDA) effect size (LEfSe) analysis was used to detect species abundance data between groups by the rank sum test to detect different species within different groups, and the magnitude of the effects of the different species biomarkers was assessed by LDA. At the family level, we found that the biomarkers of WT-V–KO-V were *Lachnospiraceae*, *Rikenellaceae*, and *Prevotellaceae* (Fig. 3a, c), and the biomarker of KO-V–KO-T was *Rikenellaceae* (Fig. 3b, d). *Alistipes* was the genus of *Rikenellaceae*. *Rikenellaceae* is positively correlated with body fat mass (*r* = 0.419, *p* = 0.027) and fat percentage (*r* = 0.398, *p* = 0.036) (Fig. 3e, f).

### Alteration of *Lachnospiraceae* and *Akkermansiaceae* abundance in calcipotriol and iBRD9-treated Nur77 knockout mice

**Fig. 4:**
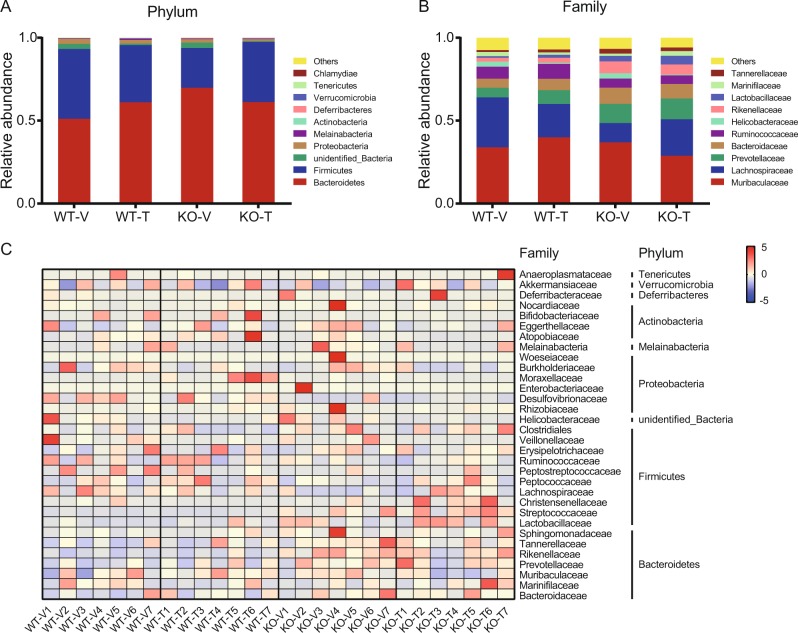
Relative abundance distribution of gut microbiota constituents at the phylum and family levels. **a** The gut microbiota constituents with the top ten greatest relative abundances at the phylum level. **b** The gut microbiota constituents with the top ten greatest relative abundances at the family level. **c** Relative abundances at the family level associated with the top ten greatest abundances at the phylum level that were altered in Nur77 knockout mice and reversed by interventions.

Figure 4a, b shows gut microbiota constituents with the top ten relative abundances at the phylum and family levels. At the phylum level, *Firmicutes* and *Bacteroidetes* together accounted for a major portion of the bacterial population in all samples (93.24–97.43%). The Nur77 knockout mouse had an increased relative abundance of *Bacteroides* that decreased after treatment (Fig. 4a). The changes in the biomarker strains *Lachnospiraceae*, *Rikenellaceae*, and *Prevotellaceae* found by LEfSe analysis are shown in Fig. 4b. The relative abundance of *Lachnospiraceae* in Nur77 knockout mice was low, and the relative abundance of *Rikenellaceae* and *Prevotellaceae* was high; the relative abundance of *Lachnospiraceae* increased after treatment, the relative abundance of *Rikenellaceae* decreased. The relative abundance of *Akkermansiaceae* in Nur77 knockout mice was low and increased after treatment (Fig. 4c).

### Calcipotriol combined with iBRD9 protected the gut intestinal barrier integrity and function of Nur77 knockout mice

**Fig. 5:**
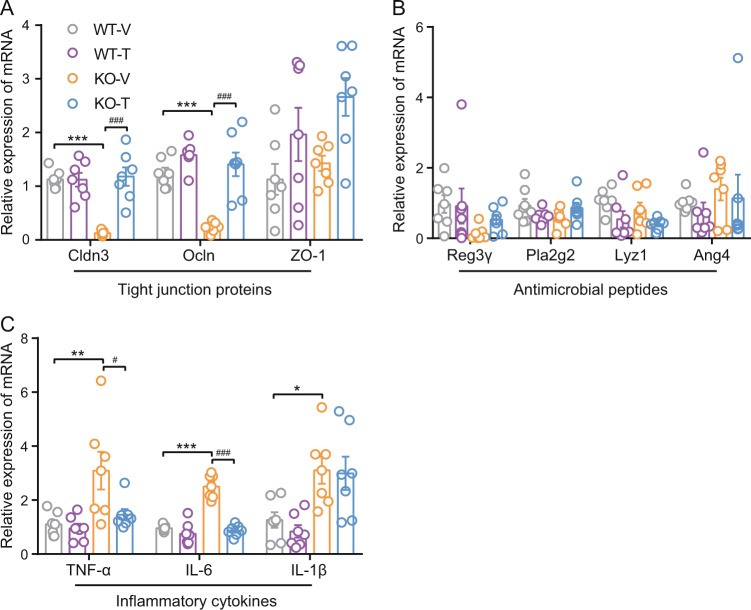
The effects of calcipotriol combined with iBRD9 on the expression of colon tight junction proteins, colon antimicrobial peptides, and colon inflammatory cytokines in Nur77 knockout mice. The mRNA expression of tight junction proteins (**a**), antimicrobial peptides (**b**), and inflammatory cytokines (**c**) in the colon (*n* = 7/group). Data are expressed as the mean ± SEM. *p < 0.05; **p < 0.01; ***p < 0.001 versus WT-V; ^#^*p* < 0.05; ^###^*p* < 0.001 versus KO-V.

Then we examined the mRNA levels of tight junction proteins, antimicrobial peptides, and inflammatory cytokines in the colon. The mRNA expression of the intestinal mucosal tight junction proteins Ocln and Cldn3 was significantly decreased in Nur77 knockout mice (Fig. 5a), and the expression of Ocln and Cldn3 was significantly increased after treatment (*p* < 0.001).

For the antibacterial peptides (Fig. 5b), namely, lysozyme C (Lyz1), regenerating islet-derived IIIγ (Reg3γ), phospholipase A2 group II (Pla2g2), and angiopoietin 4 (Ang4). There were no significant differences in antibacterial peptides between the four groups of mice.

Among the mRNA expression levels of colonic inflammatory cytokines, TNF-α, IL-6, and IL-1β were significantly increased in Nur77 knockout mice (*p* < 0.05, <0.01, or <0.001). TNF-α and IL-6 expression was significantly reduced after treatment (*p* < 0.05 or <0.001).

**Fig. 6:**
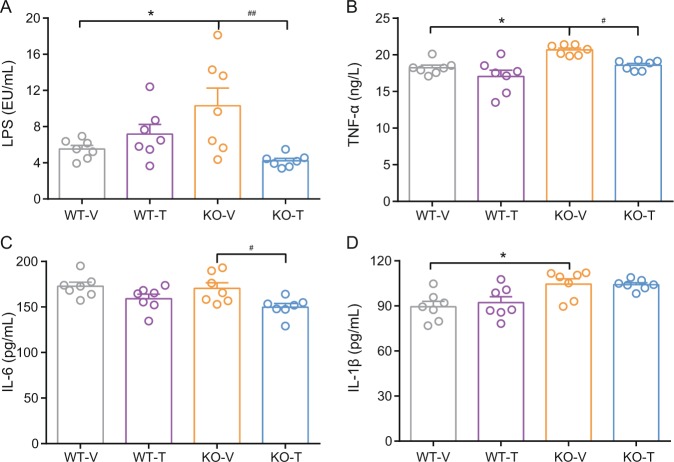
The serum levels of lipopolysaccharide and inflammatory cytokines in calcipotriol- and iBRD9-treated Nur77 knockout mice. Serum levels of lipopolysaccharide (**a**), TNF-α (**b**), IL-6 (**c**), and IL-1β (**d**) (*n* = 7/group). Data are expressed as the mean ± SEM. *p < 0.05 versus WT-V; ^#^*p* < 0.05; ^##^*p* < 0.01 versus KO-V.

To further verify changes in intestinal mucosal barrier function, we measured serum LPS and inflammatory cytokine levels in mice. The serum LPS levels of Nur77 knockout mice were significantly increased compared with those of wild-type mice and decreased significantly after treatment (*p* < 0.05 or <0.01) (Fig. 6a). The serum levels of TNF-α and IL-1β in Nur77 knockout mice increased significantly (*p* < 0.05) (Fig. 6b, d). After treatment, the TNF-α and IL-6 levels were significantly decreased (*p* < 0.05) (Fig. 6b, c).

## Discussion

In this study, we used Nur77 knockout mice and assessed the effects of calcipotriol combined with iBRD9; we found that Nur77 knockout mice showed a significant increase in body weight after 17 weeks of age, consistent with the literature [7]. After treatment with calcipotriol combined with iBRD9, the body weight of Nur77 knockout mice was significantly reduced. This change in body weight was derived from changes in body fat rather than lean weight.

We explored changes in the gut microbiota by 16S rRNA sequencing. The results showed that Nur77 deficiency had a significant effect on the gut microbiota composition, while calcipotriol combined with iBRD9 treatment had a significant effect on the composition of gut microbiota in only Nur77 knockout mice but not wild-type mice. *Bacteroidetes* and *Firmicutes* accounted for the majority of the bacterial population (93.24–97.43%), and the *Firmicutes/Bacteroidetes* ratio in Nur77 knockout mice was decrease. However, there is currently no consensus on the relationship between the *Firmicutes/Bacteroidetes* ratio and obesity [25, 26]. We were pleased to find that the Nur77 knockout mice had a low relative abundance of *Lachnospiraceae* and *Akkermansiaceae*. After calcipotriol combined with iBRD9 treatment, their abundances increased. *Lachnospiraceae* belongs to *Firmicutes* and degrades dietary fiber to produce short-chain fatty acids, which enhance intestinal barrier function by regulating tight junction proteins and mucins [27, 28]. *Akkermansiaceae* plays an important role in maintaining intestinal homeostasis, and it has been extensively studied in diseases such as obesity and inflammatory bowel disease [29–31]. In addition, we detected four groups of differentially abundant bacteria, including *Rikenellaceae*, and studies show that *Rikenellaceae* abundance increases in high-fat-fed mice [32, 33] and that *Rikenellaceae* is positively correlated with body fat percentage and fat mass (Fig. 3e, f), which is consistent with reports in humans [34].

Previous studies have shown that activation of the Nur77/RXR heterodimer is responsible for reducing monocyte-mediated inflammation in the intestine [35] and that Nur77 has an important protective effect on the development of inflammatory bowel disease [36, 37]. Therefore, it is speculated that Nur77 knockout mice may have undetected intestinal damage. Next, we examined the mRNA levels of tight junction proteins, antimicrobial peptides, and inflammatory cytokines in the colon. The mRNA expression levels of the tight junction proteins Cldn3, Ocln, and ZO-1 can reflect intestinal mucosal barrier function [30, 38–40], and the results showed that the intestinal mucosal barrier function of Nur77 knockout mice was impaired and that there was improvement after treatment with calcipotriol combined with iBRD9. The increased mRNA expression of colonic inflammatory cytokines observed in Nur77 knockout mice decreased after calcipotriol combined with iBRD9 treatment, suggesting that similar changes may occur in serum inflammation factors.

Studies in the literature have shown that increased LPS levels can induce a large number of proinflammatory responses and inflammatory cytokine release by activating Toll-like receptors 2, 4, and 5 [41, 42]. This effect may be due to the increased intestinal permeability and destruction of tight junction proteins attached to epithelial cells, increasing portal vein and systemic plasma LPS concentrations [43]. Therefore, we measured the level of serum LPS, and the serum LPS level of Nur77 knockout mice was increased significantly but decreased significantly after treatment. This finding supported the occurrence of damage of the intestinal mucosal barrier in Nur77 knockout mice and the improvement of intestinal mucosal barrier function after treatment with calcipotriol combined with iBRD9. The increase in LPS absorbed into the blood may be one of the causes of low-grade inflammation leading to obesity [44–46]. The serum levels of inflammatory factors in our experiments indicated that low-grade inflammation occurs in Nur77 knockout mice, and the level of inflammation decreased after treatment, which corresponded to LPS levels and was consistent with colon mRNA expression levels. Studies have shown that obesity is positively associated with low-grade inflammation and that obesity can be effectively alleviated by reducing inflammation [47, 48]. We applied calcipotriol combined with iBRD9 to reduce body weight by reducing the level of inflammation in Nur77 knockout mice.

Our results indicate that changes in the gut microbiota of Nur77 knockout mice contribute to obesity. Furthermore, the expression of intestinal mucosal tight junction protein-related genes is reduced, and serum LPS concentration and inflammatory factor levels are increased. Calcipotriol combined with iBRD9 can regulate the gut microbiota, improve intestinal mucosal barrier function, reduce LPS absorption into the blood, and alleviate inflammation and obesity. This study clarified the causes of low-grade inflammation and obesity in Nur77 knockout mice and demonstrated the therapeutic effect of calcipotriol combined with iBRD9 on obesity. The specific mechanism of Nur77 knockout mice gut microbiota dysbiosis, and how calcipotriol combined with iBRD9 regulates the gut microbiota have not been explored in depth, but it is a subject worth studying.

## Supplementary information

Table S1

Table S2
