# Supplementary material for: Calcipotriol and iBRD9 reduce obesity in Nur77 knockout mice by regulating the gut microbiota, improving intestinal mucosal barrier function
Source: Int J Obes (Lond). 2020 Mar 17;44(5):1052–61. doi: 10.1038/s41366-020-0564-0 (PMC7188666; doi:10.1038/s41366-020-0564-0)
Supplement: Supplementary file 1 — Table S1 [file 41366_2020_564_MOESM1_ESM.docx]

Table S1. Mouse primers used in this study.

| Gene | Forward primer (5'-3') | Reverse primer (5'-3') |
| --- | --- | --- |
| Cldn3 | TCATCGGCAGCAGCATCATCAC | ACGATGGTGATCTTGGCCTTGG |
| Ocln | ATGTCCGGCCGATGCTCTC | TTTGGCTGCTCTTGGGTCTGTAT |
| ZO-1 | TTTTTGACAGGGGGAGTGG | TGCTGCAGAGGTCAAAGTTCAAG |
| Reg3g | TTCCTGTCCTCCATGATCAAA | CATCCACCTCTGTTGGGTTC |
| Pla2g2 | AGGATTCCCCCAAGGATGCCAC | CAGCCGTTTCTGACAGGAGTTCTGG |
| Lyz1 | GCCAAGGTCTACAATCGTTGTGAGTTG | CAGTCAGCCAGCTTGACACCACG |
| Ang4 | CTCTGGCTCAGAATGTAAGGTACGA | GAAATCTTTAAAGGCTCGGTACCC |
| IL-6 | TAGTCCTTCCTACCCCAATTTCC | TTGGTCCTTAGCCACTCCTTC |
| IL-1β | GCAACTGTTCCTGAACTCAACT | ATCTTTTGGGGTCCGTCAACT |
| TNF-α | CTGGATGTCAATCAACAATGGGA | ACTAGGGTGTGAGTGTTTTCTGT |
| GAPDH | AGGTCGGTGTGAACGGATTTG | TGTAGACCATGTAGTTGAGGTCA |
