# Supplementary material for: Calcipotriol and iBRD9 reduce obesity in Nur77 knockout mice by regulating the gut microbiota, improving intestinal mucosal barrier function
Source: Int J Obes (Lond). 2020 Mar 17;44(5):1052–61. doi: 10.1038/s41366-020-0564-0 (PMC7188666; doi:10.1038/s41366-020-0564-0)
Supplement: Supplementary file 2 — Table S2 [file 41366_2020_564_MOESM2_ESM.docx]

Table S2 Serum biochemical parameters of calcipotriol and iBRD9-treated Nur77 knockout mice.

| Parameters | WT-V | WT-T | KO-V | KO-T | *P* value |
| --- | --- | --- | --- | --- | --- |
| FBG (mmol/L) | 8.27±0.94 | 8.06±1.14 | 7.67±1.29 | 7.53±0.81 | 0.544 |
| Leptin (pg/mL) | 373.99±35.88 | 342.79±51.72 | 413.32±34.76* | 351.83±40.29# | 0.018 |
| TC (mmol/L) | 3.88±0.67 | 4.18±0.91 | 4.44±0.74 | 4.02±0.41 | 0.491 |
| TG (mmol/L) | 0.60±0.21 | 0.42±0.35 | 0.33±0.05 | 0.34±0.09 | 0.091 |
| Ca (mmol/L) | 2.09±0.08 | 2.11±0.04 | 2.05±0.12 | 2.02±0.03 | 0.127 |
| P (mmol/L) | 1.15±0.15 | 1.25±0.10 | 1.29±0.07 | 1.26±0.10 | 0.094 |

Data are expressed as the mean ± SEM. **p* < 0.05 versus WT-V; # *p* < 0.05 versus KO-V.
